# An adaptive biomarker strategy clinical trial design

**DOI:** 10.1186/1745-6215-14-S1-O103

**Published:** 2013-11-29

**Authors:** James Wason, Nigel Stallard, Janet Dunn, Rob Stein

**Affiliations:** 1 MRC Biostatistics Unit Hub for Trials Methodology Research, Cambridge, UK; 2 Warwick Medical School, University of Warwick, Coventry, UK; 3 Warwick Clinical Trials Unit, Warwick Medical School, University of Warwick, Coventry, UK; 4 Department of Oncology, UCL Hospitals, London, UK

## 

The Biomarker Strategy Design has been proposed for trials assessing the value of a biomarker in guiding treatment in oncology. In such trials, patients are randomised to a control arm, receiving the standard chemotherapy treatment, or a biomarker-directed treatment arm, when biomarker status is used to guide treatment. The randomised groups are then compared, often using a non-inferiority test to assess whether the biomarker-guided therapy, limiting potentially toxic chemotherapy to a subgroup of patients, is at least as effective as universal chemotherapy.

Motivated by a current trial of biomarker guided adjuvant chemotherapy in breast cancer, we consider an adaptive version of this design in which two biomarkers are assessed. The trial is conducted in two stages. In the first stage, patients in the test-guided arm receive both a standard and a new test, with the standard test guiding treatment. An analysis comparing test results from this stage is then used to choose the test to use in the remainder of the trial, using the new test if the results for the two tests are sufficiently similar.

We used the delta method to approximate the joint distribution of the results from the two stages, showing that in practical situations the correlation between these is near zero. First stage results can therefore be used to adapt the trial without error rate inflation. We also show that for an appropriately chosen decision rule there can be considerable cost gains with only a small loss in power.

